# Paced Breathing Increases the Redundancy of Cardiorespiratory Control in Healthy Individuals and Chronic Heart Failure Patients

**DOI:** 10.3390/e20120949

**Published:** 2018-12-10

**Authors:** Alberto Porta, Roberto Maestri, Vlasta Bari, Beatrice De Maria, Beatrice Cairo, Emanuele Vaini, Maria Teresa La Rovere, Gian Domenico Pinna

**Affiliations:** 1 Department of Biomedical Sciences for Health, University of Milan, 20097 Milan, Italy; 2 Department of Cardiothoracic, Vascular Anesthesia and Intensive Care, IRCCS Policlinico San Donato, San Donato Milanese, 20097 Milan, Italy; 3 Department of Biomedical Engineering, IRCCS Istituti Clinici Scientifici Maugeri, 27040 Montescano, Italy; 4 IRCCS Istituti Clinici Scientifici Maugeri, 20097 Milan, Italy; 5 Department of Cardiological Rehabilitation, IRCCS Istituti Clinici Scientifici Maugeri, 27040 Montescano, Italy

**Keywords:** generalized mutual information, interaction self-information, bivariate dynamical stochastic system, linear regression model, partial information decomposition, synergy, cardiorespiratory coupling, heart rate variability, autonomic nervous system

## Abstract

Synergy and redundancy are concepts that suggest, respectively, adaptability and fault tolerance of systems with complex behavior. This study computes redundancy/synergy in bivariate systems formed by a target X and a driver Y according to the predictive information decomposition approach and partial information decomposition framework based on the minimal mutual information principle. The two approaches assess the redundancy/synergy of past of X and Y in reducing the uncertainty of the current state of X. The methods were applied to evaluate the interactions between heart and respiration in healthy young subjects (n = 19) during controlled breathing at 10, 15 and 20 breaths/minute and in two groups of chronic heart failure patients during paced respiration at 6 (n = 9) and 15 (n = 20) breaths/minutes from spontaneous beat-to-beat fluctuations of heart period and respiratory signal. Both methods suggested that slowing respiratory rate below the spontaneous frequency increases redundancy of cardiorespiratory control in both healthy and pathological groups, thus possibly improving fault tolerance of the cardiorespiratory control. The two methods provide markers complementary to respiratory sinus arrhythmia and the strength of the linear coupling between heart period variability and respiration in describing the physiology of the cardiorespiratory reflex suitable to be exploited in various pathophysiological settings.

## 1. Introduction

Quantifying redundancy/synergy among interacting systems is one of the most relevant challenges of signal processing because it is believed that these concepts are linked, respectively, to fault tolerance and adaptability typical of complex and self-organized systems [1]. Increasing redundancy means to improve fault tolerance because robust adjustments of the target state are preserved in presence of partial drop of the input-output connections given that the separate influences of sources are more powerful than their joint action in governing the target behavior. Increasing synergy means improving adaptability because the contemporaneous action of sources produces nontrivial effects on the target stronger than those resulting from the separate influences of the sources, thus enriching the variety of the target responses to inputs and favoring the emergence of unexpected behaviors. The quantification of redundancy/synergy is usually performed in a multivariate context (i.e., higher than bivariate) describing interactions among multiple components of the same system or different systems forming a network and is mainly limited to the assessment of redundant and synergistic effects of sources in transferring information into the target [2,3,4,5,6,7,8]. However, synergy and redundancy are concepts that are not indissolubly linked to multivariate systems and information transfer. Indeed, redundant and synergistic terms can arise from the interactions among any set formed by three stochastic variables [9,10,11,12,13]. Thereby, a redundant/synergistic term can be found in bivariate dynamical systems when the pasts of the target and driver influence the present of the target [14,15]. This possibility raises the question whether redundancy/synergy parameters computed in bivariate applications have some practical value. Cardiorespiratory interactions [16] are mostly studied in a bivariate context by considering the beat-to-beat spontaneous changes of heart period (HP) associated to respiration (R) via the evaluation of the amplitude of HP variations at the respiratory rate, usually referred to as respiratory sinus arrhythmia [17], the strength of HP-R coupling via information-domain, frequency-domain or model-based approaches [18,19,20] and the degree of phase synchronization between heartbeat and R [21,22,23,24,25,26]. The importance of the evaluation of cardiorespiratory interactions lies in their modifications with the state of the autonomic nervous system, sleep, aging and pathology [16,17,18,19,20,21,22,23,24,25,26].

In this study we hypothesize that parameters describing redundancy and synergy in the bivariate context of the cardiorespiratory interactions can provide relevant pathophysiological information. To test this hypothesis, we compute redundant/synergistic terms between the spontaneous fluctuations of HP and R via a model-based approach, according to two frameworks based on predictive and partial information decomposition strategies respectively, in protocols slowing breathing rate in healthy young humans [27] and chronic heart failure (CHF) patients [28]. More traditional indexes assessing cardiorespiratory interactions are computed to check the additional information provided by redundancy/synergy markers.

## 2. Quantifying Redundancy/Synergy in Bivariate Stochastic Systems

### 2.1. Notation and Preliminaries

Let us consider a bivariate dynamical system composed by two possibly intertwined systems X and Y. Let us assume that the evolution of X and Y is described by the bivariate dynamical stochastic process ***X*** = {*X*,*Y*}. We indicate with *X_n_* and *Y_n_* the discrete stochastic variables describing the present of *X* and *Y* and with $X_{n}^{-}=\left[X_{n-1}X_{n-2}\cdots \right]$ and $Y_{n}^{-}=\left[Y_{n-1}Y_{n-2}\cdots \right]$ the discrete vector stochastic variables describing the past of *X* and *Y*, respectively The discrete stochastic variables *X_n_*, *Y_n_*, $X_{n}^{-}$ and $Y_{n}^{-}$ assume values in the sets $A_{X_{n}}$, $A_{Y_{n}}$, $A_{X_{n}^{-}}$ and $A_{Y_{n}^{-}}$, respectively. We denote with *p*(*x_n_*), *p*(*y_n_*), $p\left(x_{n}^{-}\right)$ and $p\left(y_{n}^{-}\right)$ the probability of observing *X_n_* = *x_n_*, *Y_n_* = *y_n_*, $X_{n}^{-}=x_{n}^{-}$ and $Y_{n}^{-}=y_{n}^{-}$ respectively and with $p\left(x_{n},x_{n}^{-}\right)$, $p\left(y_{n},y_{n}^{-}\right)$, $p\left(x_{n}^{-},y_{n}^{-}\right)$ and $p\left(x_{n},x_{n}^{-},y_{n}^{-}\right)$ the joint probabilities of observing, respectively, *X_n_* = *x_n_* and $X_{n}^{-}=x_{n}^{-}$, *Y_n_* = *y_n_* and $Y_{n}^{-}=y_{n}^{-}$, $X_{n}^{-}=x_{n}^{-}$ and $Y_{n}^{-}=y_{n}^{-}$, *X_n_* = *x_n_* and $X_{n}^{-}=x_{n}^{-}$ and $Y_{n}^{-}=y_{n}^{-}$. The Bayes rule allows the computation of conditional probabilities $p\left(x_{n}|y_{n}^{-}\right)=p\left(x_{n},y_{n}^{-}\right)/p\left(y_{n}^{-}\right)$, and $p\left(x_{n}|x_{n}^{-},y_{n}^{-}\right)=p\left(x_{n},x_{n}^{-},y_{n}^{-}\right)/p\left(x_{n}^{-},y_{n}^{-}\right)$. In the following we will consider X as the target system and Y as the driver system and the information-theoretic quantities will be assessed in the causal direction from Y to X.

### 2.2. Basic Information-Theoretic Quantities Contributing to Interaction Self-Information in Bivariate Stochastic Systems

In the following we recall the definitions of some quantities necessary to compute the interaction self-information in bivariate stochastic systems [29]: (i) the Shannon entropy of *X* [30]:

(1) $$H_{X}\left(X_{n}\right)=-\underset{x_{n}\in A_{X_{n}}}{\sum }p\left(x_{n}\right)\cdot log\;p\left(x_{n}\right)$$

where log is the natural logarithm, measures the amount of information carried by the present of *X*; (ii) the Shannon entropy of $X^{-}$ [30]:

(2) $$H_{X^{-}}\left(X_{n}^{-}\right)=-\underset{x_{n}^{-}\in A_{X_{n}^{-}}}{\sum }p\left(x_{n}^{-}\right)\cdot log\;p\left(x_{n}^{-}\right)$$

quantifies the amount of information carried by the past of *X*; (iii) the Shannon entropy of $Y^{-}$ [30]:

(3) $$H_{Y^{-}}\left(Y_{n}^{-}\right)=-\underset{y_{n}^{-}\in A_{Y_{n}^{-}}}{\sum }p\left(y_{n}^{-}\right)\cdot log\;p\left(y_{n}^{-}\right)$$

quantifies the amount of information carried by the past of *Y*; (iv) the self-entropy of *X* [27,31,32]:

(4) $$S_{X}\left(X_{n}\right)=\underset{x_{n}\in A_{X_{n}},x_{n}^{-}\in A_{X_{n}^{-}}}{\sum }p\left(x_{n},x_{n}^{-}\right)\cdot log\frac{p\left(x_{n},x_{n}^{-}\right)}{p\left(x_{n}\right)\cdot p\left(x_{n}^{-}\right)}$$

measures the amount of information carried by *X_n_* that can be resolved by $X_{n}^{-}$, usually referred to as information stored into *X*; (v) the conditional self-entropy of *X* given *Y* [29,33]:

(5) $$S_{X|Y}\left(X_{n}\right)=\underset{x_{n}\in A_{X_{n}},x_{n}^{-}\in A_{X_{n}^{-}},y_{n}^{-}\in A_{Y_{n}^{-}}}{\sum }p\left(x_{n},x_{n}^{-},y_{n}^{-}\right)\cdot log\frac{p(x_{n},x_{n}^{-}|y_{n}^{-})}{p(x_{n}|y_{n}^{-})\cdot p(x_{n}^{-}|y_{n}^{-})}$$

quantities the amount of information carried by *X_n_* that can be resolved by $X_{n}^{-}$ above and beyond that can be obtained from $Y_{n}^{-}$; (vi) the cross-entropy from *Y* to *X* [34]:

(6) $$C_{Y\to X}\left(X_{n}\right)=\underset{x_{n}\in A_{X_{n}},y_{n}^{-}\in A_{Y_{n}^{-}}}{\sum }p\left(x_{n},y_{n}^{-}\right)\cdot log\frac{p\left(x_{n},y_{n}^{-}\right)}{p\left(x_{n}\right)\cdot p\left(y_{n}^{-}\right)}$$

measures the amount of information carried by *X_n_* that can be derived from $Y_{n}^{-}$, usually referred to as the cross-information from *Y* to *X*; (vii) the transfer entropy from *Y* to *X* [35]:

(7) $$T_{Y\to X}\left(X_{n}\right)=\underset{x_{n}\in A_{X_{n}},x_{n}^{-}\in A_{X_{n}^{-}},y_{n}^{-}\in A_{Y_{n}^{-}}}{\sum }p\left(x_{n},x_{n}^{-},y_{n}^{-}\right)\cdot log\frac{p(x_{n},y_{n}^{-}|x_{n}^{-})}{p(x_{n}|x_{n}^{-})\cdot p(y_{n}^{-}|x_{n}^{-})}$$

quantities the amount of information carried by *X_n_* that can be resolved by $Y_{n}^{-}$ above and beyond that can be obtained from $X_{n}^{-}$. From Equations (4)–(7) it can be easily recognized that $S_{X}\left(X_{n}\right)$, $S_{X|Y}\left(X_{n}\right)$, $C_{Y\to X}\left(X_{n}\right)$ and $T_{Y\to X}\left(X_{n}\right)$ are mutual information (MI) or conditional MI (CMI) functions. More specifically, $S_{X}\left(X_{n}\right)=MI\left(X_{n};X_{n}^{-}\right)$, $S_{X|Y}\left(X_{n}\right)=CMI(X_{n};X_{n}^{-}|Y_{n}^{-})$, $C_{Y\to X}\left(X_{n}\right)=MI\left(X_{n};Y_{n}^{-}\right)$ and $T_{Y\to X}\left(X_{n}\right)=CMI(X_{n};Y_{n}^{-}|X_{n}^{-})$. Being MI and CMI between two stochastic variables, they are all nonnegative quantities. The mnemonic Venn diagram of the information-theoretic measures reported in (Equations (1)–(7)) is given in Figure 1. 

An additional useful information-theoretic quantity is the predictive information of *X* in ***X*** [29]:

(8) $$P_{X}\left(X_{n}\right)=\underset{x_{n}\in A_{X_{n}},x_{n}^{-}\in A_{X_{n}^{-}},y_{n}^{-}\in A_{Y_{n}^{-}}}{\sum }p\left(x_{n},x_{n}^{-},y_{n}^{-}\right)\cdot log\frac{p\left(x_{n},x_{n}^{-},y_{n}^{-}\right)}{p\left(x_{n}\right)\cdot p\left(x_{n}^{-},y_{n}^{-}\right)}$$

measuring the amount of information of the present state *X_n_* that can be resolved from the joint observation of the past of *X* and *Y* computed as the $MI\left(X_{n};X_{n}^{-},Y_{n}^{-}\right)$. According to [29]:

(9) $$P_{X}\left(X_{n}\right)=S_{X|Y}\left(X_{n}\right)+C_{Y\to X}\left(X_{n}\right)=S_{X}\left(X_{n}\right)+T_{Y\to X}\left(X_{n}\right)$$

$P_{X}\left(X_{n}\right)$ is nonnegative given that it is sum of nonnegative quantities.

### 2.3. Generalized MI and its Link with Interaction Self-Information in Bivariate Stochastic Systems

In ***X*** = {*X*,*Y*} the generalized MI (GMI), $GMI\left(X_{n};X_{n}^{-};Y_{n}^{-}\right)$, is related to how the pasts of *X* and *Y* interact with each other in explaining the information carried by the current state *X_n_*. $GMI\left(X_{n};X_{n}^{-};Y_{n}^{-}\right)$ is given by (Figure 1a,c):

(10) $$GMI\left(X_{n};X_{n}^{-};Y_{n}^{-}\right)=S_{X}\left(X_{n}\right)-S_{X|Y}\left(X_{n}\right)$$

**Figure 2:**
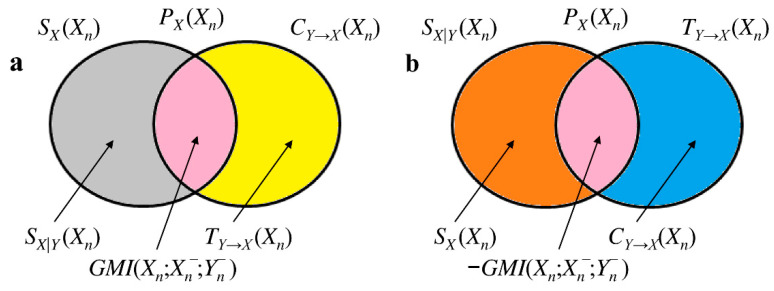
Mnemonic Venn diagrams of information-theoretic quantities useful to characterize $GMI\left(X_{n};X_{n}^{-};Y_{n}^{-}\right)$ of the bivariate process ***X*** = {*X*,*Y*}. Positive $GMI\left(X_{n};X_{n}^{-};Y_{n}^{-}\right)$ is depicted in (**a**) with $S_{X}\left(X_{n}\right)=GMI\left(X_{n};X_{n}^{-};Y_{n}^{-}\right)+S_{X|Y}\left(X_{n}\right)$ and $C_{Y\to X}\left(X_{n}\right)=GMI\left(X_{n};X_{n}^{-};Y_{n}^{-}\right)+T_{Y\to X}\left(X_{n}\right)$. Negative $GMI\left(X_{n};X_{n}^{-};Y_{n}^{-}\right)$ is depicted in (**b**) with $S_{X|Y}\left(X_{n}\right)=-GMI\left(X_{n};X_{n}^{-};Y_{n}^{-}\right)+S_{X}\left(X_{n}\right)$ and $T_{Y\to X}\left(X_{n}\right)=-GMI\left(X_{n};X_{n}^{-};Y_{n}^{-}\right)+C_{Y\to X}\left(X_{n}\right)$. The color code is the same adopted in Figure 1. All the depicted areas are positive.

$GMI\left(X_{n};X_{n}^{-};Y_{n}^{-}\right)$ measures the influence of the past of *Y* on the information shared between *X_n_* and $X_{n}^{-}$. $GMI\left(X_{n};X_{n}^{-};Y_{n}^{-}\right)=0$ when $S_{X}\left(X_{n}\right)=S_{X|Y}\left(X_{n}\right)$, namely when the knowledge of the past of *Y* is useless in explaining the dependency of *X* on $X_{n}^{-}$. $GMI\left(X_{n};X_{n}^{-};Y_{n}^{-}\right)=S_{X}\left(X_{n}\right)$ when $S_{X|Y}\left(X_{n}\right)=0$, namely when the information stored into *X* is completely explained by $Y_{n}^{-}$. Positive $GMI\left(X_{n};X_{n}^{-};Y_{n}^{-}\right)$ indicates that past of *Y* inhibits the information storage into *X* because it accounts for, or explains part of, the dependency of *X* on $X_{n}^{-}$. As a consequence we say that the past of *Y* contributes redundantly to the information sharing between *X* and $X_{n}^{-}$. When $GMI\left(X_{n};X_{n}^{-};Y_{n}^{-}\right)>0$,

(11) $$S_{X}\left(X_{n}\right)=GMI\left(X_{n};X_{n}^{-};Y_{n}^{-}\right)+S_{X|Y}\left(X_{n}\right)$$

holds (Figure 2a). Negative $GMI\left(X_{n};X_{n}^{-};Y_{n}^{-}\right)$ suggests that $Y_{n}^{-}$ enhances the correlation between *X* and $X_{n}^{-}$ and, thus, contributes synergistically to the information exchange between *X* and $X_{n}^{-}$. When $GMI\left(X_{n};X_{n}^{-};Y_{n}^{-}\right)<0$:

(12) $$S_{X|Y}\left(X_{n}\right)=-GMI\left(X_{n};X_{n}^{-};Y_{n}^{-}\right)+S_{X}\left(X_{n}\right)$$

holds (Figure 2b). Since:

(13) $$GMI\left(X_{n};X_{n}^{-};Y_{n}^{-}\right)=C_{Y\to X}\left(X_{n}\right)-T_{Y\to X}\left(X_{n}\right)$$

holds as well (Figure 1b,c), $GMI\left(X_{n};X_{n}^{-};Y_{n}^{-}\right)$ equivalently measures the influence of the past of *X* on the information sharing between *X_n_* and $Y_{n}^{-}$. $GMI\left(X_{n};X_{n}^{-};Y_{n}^{-}\right)=0$ when $C_{Y\to X}\left(X_{n}\right)=T_{Y\to X}\left(X_{n}\right)$, namely when the knowledge of the past of *X* is useless in explaining the dependency of *X* on $Y_{n}^{-}$. $GMI\left(X_{n};X_{n}^{-};Y_{n}^{-}\right)=C_{Y\to X}\left(X_{n}\right)$ when $T_{Y\to X}\left(X_{n}\right)=0$, namely when the cross-information from *Y* to *X* completely explained by $X_{n}^{-}$. Positive $GMI\left(X_{n};X_{n}^{-};Y_{n}^{-}\right)$ indicates that past of *X* inhibits the cross-information from *Y* to *X* because it accounts for, or explains part of, the dependency of *X* on $Y_{n}^{-}$. Thereby, we deduce that the past of *X* contributes redundantly to the information sharing between *X* and $Y_{n}^{-}$. When $GMI\left(X_{n};X_{n}^{-};Y_{n}^{-}\right)>0$,

(14) $$C_{Y\to X}\left(X_{n}\right)=GMI\left(X_{n};X_{n}^{-};Y_{n}^{-}\right)+T_{Y\to X}\left(X_{n}\right)$$

holds (Figure 2a). Negative $GMI\left(X_{n};X_{n}^{-};Y_{n}^{-}\right)$ suggests that $X_{n}^{-}$ enhances the correlation between *X* and $Y_{n}^{-}$, and, thus, contributes synergistically to the information exchange between *X* and $Y_{n}^{-}$. When $GMI\left(X_{n};X_{n}^{-};Y_{n}^{-}\right)<0$,

(15) $$T_{Y\to X}\left(X_{n}\right)=-GMI\left(X_{n};X_{n}^{-};Y_{n}^{-}\right)+C_{Y\to X}\left(X_{n}\right)$$

holds (Figure 2b). We define the bivariate interaction self-information as $I_{X,Y}^{X}\left(X_{n}\right)=-GMI\left(X_{n};X_{n}^{-};Y_{n}^{-}\right)$. This relation links more intuitively positive $I_{X,Y}^{X}\left(X_{n}\right)$ to the synergistic ability of the past of *Y* in enhancing the information storage in *X* or, equivalently, the synergistic ability of the past of *X* in enhancing the cross-information from *Y* to *X*, and negative $I_{X,Y}^{X}\left(X_{n}\right)$ to the redundant capacity of the past of *Y* in inhibiting the information storage in *X* or, equivalently, the redundant capacity of the past of *X* in inhibiting the cross-information from *Y* to *X*. From Figure 1 it can be deduced that:

(16) $$P_{X}\left(X_{n}\right)=S_{X|Y}\left(X_{n}\right)+GMI\left(X_{n};X_{n}^{-};Y_{n}^{-}\right)+T_{Y\to X}\left(X_{n}\right)=S_{X}\left(X_{n}\right)+I_{X,Y}^{X}\left(X_{n}\right)+C_{Y\to X}\left(X_{n}\right)$$

Therefore, when the sum of the separate contributions of the past of *X* and the past of *Y* to the information carried by the current state *X_n_*, represented by $S_{X}\left(X_{n}\right)$ and $C_{Y\to X}\left(X_{n}\right)$ respectively, is smaller than their joint contribution measured by the $P_{X}\left(X_{n}\right)$, we observe synergy (i.e., $I_{X,Y}^{X}\left(X_{n}\right)>0$). The opposite is observed in presence of redundancy.

### 2.4. Separating the Contributions of Redundancy and Synergy in Bivariate Interaction Self-Information

The limitation of the approach to the computation of interaction self-information completely framed within the Shannon information theory is that $I_{X,Y}^{X}\left(X_{n}\right)$ is the net balance between synergy and redundancy [7,8] and the computation of synergy and redundancy as separate nonnegative quantities is prevented. Partial information decomposition approach extends the traditional relations derived according to the rules of Shannon information theory, basically Equations (10), (13) and (16), by adding an additional axiom that depends on the idea underlying what synergy and redundancy should really represent while assuring their nonnegativity and symmetry to the permutation of $X_{n}^{-}$ with $Y_{n}^{-}$ [9,10,11]. Among the possible solutions within the partial information decomposition framework we selected the one proposed in [9] and referred to as minimum MI. This method computes redundancy of $X_{n}^{-}$ and $Y_{n}^{-}$ to *X_n_* as:

(17) $$R_{X}\left(X_{n};X_{n}^{-},Y_{n}^{-}\right)=min[MI\left(X_{n};X_{n}^{-}\right),MI\left(X_{n};Y_{n}^{-}\right)]$$

where min takes the minimum of the two arguments, and uses the net balance between synergy and redundancy, namely:

(18) $$I_{X,Y}^{X}\left(X_{n}\right)=S_{X}\left(X_{n};X_{n}^{-},Y_{n}^{-}\right)-R_{X}\left(X_{n};X_{n}^{-},Y_{n}^{-}\right)$$

to compute $S_{X}\left(X_{n};X_{n}^{-},Y_{n}^{-}\right)$.

### 2.5. Estimation of the Bivariate Interaction Self-Information

The estimation of the bivariate interaction self-information is carried out via a model-based parametric linear approach under the hypotheses of stationarity and Gaussian distribution of *X* and *Y*. The zero mean process *X* with variance *λ*^2^ is modeled as an autoregressive (AR) model with exogenous (X) input (ARX) describing the current state *X_n_* as the linear combination of *p* past states of the same process weighted by the coefficients *a_k_*, with *k* = 1, …, *p* plus the linear combination of *p* − *τ* + 1 past states of *Y*, including possibly the current one if *τ* = 0, weighted by the coefficients *b_k_*, with *k* = *τ*, …, *p* plus a random unpredictable portion *W_n_*, being the sampling of a Gaussian white noise *W* with zero mean and variance $\lambda_{ARX}^{2}$, namely:

(19) $$X_{n}=\overset{p}{\underset{k=1}{\sum }}a_{k}\cdot X_{n-k}+\overset{p}{\underset{k=\tau }{\sum }}b_{k}\cdot Y_{n-k}+W_{n}$$

where *p* is the model order and *τ* is the delay of the actions from *Y* to *X*. The ARX model can be reduced to an AR model of *X* whether the linear regression of *X_n_* on the past, and possibly present, of *Y* is excluded and the variance of the unpredictable part is $\lambda_{AR}^{2}$, and to an X model of *X* whether the linear regression of *X_n_* on the past of *X* is excluded and the variance of the unpredictable part is $\lambda_{X}^{2}$. According to [29,33,36], under the hypotheses of stationarity and Gaussianity the terms present in Equation (10) can be computed as:

(20) $$S_{X}\left(X_{n}\right)=\frac{1}{2}log\frac{\lambda^{2}}{\lambda_{AR}^{2}},and\;S_{X|Y}\left(X_{n}\right)=\frac{1}{2}log\frac{\lambda_{X}^{2}}{\lambda_{ARX}^{2}}$$

while the terms present in (13) can be calculated as:

(21) $$C_{Y\to X}\left(X_{n}\right)=\frac{1}{2}log\frac{\lambda^{2}}{\lambda_{X}^{2}},\;and\;T_{Y\to X}\left(X_{n}\right)=\frac{1}{2}log\frac{\lambda_{AR}^{2}}{\lambda_{ARX}^{2}}$$

thus leading to:

(22) $$I_{X,Y}^{X}\left(X_{n}\right)=\frac{1}{2}log\frac{\lambda_{X}^{2}\cdot \lambda_{AR}^{2}}{\lambda^{2}\cdot \lambda_{ARX}^{2}}$$

$S_{X}\left(X_{n};X_{n}^{-},Y_{n}^{-}\right)$ and $R_{X}\left(X_{n};X_{n}^{-},Y_{n}^{-}\right)$ are computed according to Equations (17) and (18) with $MI\left(X_{n};X_{n}^{-}\right)$ and $MI\left(X_{n};Y_{n}^{-}\right)$ given by the first part of Equations (20) and (21), respectively, and $I_{X,Y}^{X}\left(X_{n}\right)$ given by Equation (22).

## 3. Experimental Protocols

### 3.1. Paced Breathing in Healthy Young Subjects

The data belong to an historical database designed to evaluate the relationship between complexity of the cardiac autonomic control and breathing rate in healthy humans [27]. We make reference to [27] for a detailed description of the population and experimental setup. Briefly, after a period of stabilization we recorded surface electrocardiogram (II lead) in 19 healthy young humans (aged from 27 to 35 years, median = 31 years; percentage of males: 42%) and respiratory flow via a nasal thermistor (Marazza, Monza, Italy). Signals were sampled at 300 Hz. The experimental protocol included four sessions: the first session at rest in supine position with spontaneous respiration (SR) was followed by three sessions of controlled respiration (CR) in random order with the subject lying in supine position and controlling his/her breathing rate according to a metronome at 10, 15, and 20 breaths/min (CR10, CR15, and CR20). All sessions lasted 10 min. All the subjects were familiar with the paced breathing procedure and were able to follow without particular discomfort the pace given by the metronome. The protocol adhered to the principles of the Declaration of Helsinki. The ethical review boards of the “L. Sacco” Hospital, Milan, Italy, approved the protocol. Written informed consent was obtained from all subjects.

### 3.2. Paced Breathing in CHF Patients

Twenty CHF patients enrolled in the paced breathing protocol were studied in the morning in supine position (age: 56.1 ± 10.8 years, percentage of males: 85%; left ventricular ejection fraction: 35.6 ± 11.3, New York Heart Association class: 2.4 ± 0.7, 90% in class II and III, mean ± standard deviation). The experimental protocol was carried out at IRCCS Istituti Clinici Scientifici Maugeri, Montescano, Italy, and comprised: (1) instrumentation, patient’s familiarization with paced breathing and signal stabilization (about 20 min); (2) 8 mins recording of electrocardiogram and lung volume (Respitrace Plus, Non-Invasive Monitoring Systems, city, state abbrev if USA, country) during spontaneous breathing; (3) 8 min recording of the same signals during CR15. To perform paced breathing, subjects were asked to follow a played back human voice recording indicating inspiratory and expiratory phases. Signals were sampled at 250 Hz. The protocol adhered to the principles of the Declaration of Helsinki. The ethical review board of the IRCCS Istituti Clinici Scientifici Maugeri, Montescano, Italy, approved the protocol. Written informed consent was obtained from all patients.

### 3.3. Slow Breathing in CHF Patients

Nine CHF patients (age: 57.4 ± 5.2 years, percentage of males: 78%, left ventricular ejection fraction: 26.1 ± 4.3, New York Heart Association class: 2.4 ± 0.7, 89% in class II and III, mean ± standard deviation) were enrolled in the device-guided slow breathing protocol by the use of the RESPeRATE^®^ device (InterCure, Lod, city, Israel). This device guides the user interactively and progressively slowing their breathing at a controlled rate of around 6 breaths/minute (CR6). It consists of a control box containing a microprocessor, a belt-type respiration sensor and headphones. At the beginning, the device analyses the breathing rate and pattern and creates a personalized melody comprising two distinct tones, one for inhalation and one for exhalation. As the patient synchronizes inhalation and exhalation with the tones, the device gradually increases the relative duration of the exhalation tone thereby slowing the breathing rate till the target frequency is reached. This breathing guiding process requires minimal conscious effort, as the device adapts itself automatically to the ability of its user to follow the inhalation/exhalation guiding tones without dictating any predetermined breathing pattern. Signals were sampled at 250 Hz. The protocol adhered to the principles of the Declaration of Helsinki. The ethical review board of the IRCCS Istituti Clinici Scientifici Maugeri, Montescano, Italy, approved the protocol. Written informed consent was obtained from all patients.

### 3.4. Extraction of Beat-to-Beat HP Variability, R Series and Breathing Rate

HP was approximated as the time distance between two consecutive R-wave peaks detected on the electrocardiogram via a traditional method based on a threshold on the first derivative. Jitters in locating the R-wave peak were minimized using parabolic interpolation. R signal was downsampled at the first R-wave peak defining the onset of the current HP. 

All R-wave peak detections were carefully checked to avoid erroneous identifications or missed beats. Missed R-wave peaks were manually inserted and erroneous detections were fixed. HP and R values directly connected with isolated ectopic beats or more complex arrhythmic episodes were linearly interpolated starting from the closest values that were not influenced by the occurrence of the non-sinus beats. The percentage of corrections was below 5%. The R rate, indicated as f_R_, was extracted as the frequency of the dominant peak of the R series power spectral density via a parametric approach modeling the series as a realization of an AR process [37]. The AR model order was optimized in the range from 8 to 14 via the Akaike’s figure of merit and the coefficients of the model as well as the variance of the white noise were identified via the Levinson-Durbin recursive method [37].

### 3.5. Computing Interaction Self-Information from Beat-to-Beat HP Variability and R Series

According to short-term analysis of cardiorespiratory control short R and HP series (about 250 consecutive values) were taken as realizations of the process *Y* and *X*, respectively. The delay *τ* from R to HP was set to 0 beats to allow the description of the fast vagal action of cardiopulmonary reflexes [38,39]. Therefore, the cross-regression of HP on R had *p* + 1 coefficients, while the auto-regression of HP on its own past values had *p* coefficients. After normalizing the R and HP series to have zero mean and unit variance by subtracting the mean and by dividing the result by the standard deviation, the coefficients of the ARX, AR and X models were identified via traditional least squares approach and Cholesky decomposition method [40,41]. The model order *p* was optimized in the range from 4 to 16 according to the Akaike’s figure of merit for bivariate processes [42] over the ARX model. AR and X models were separately identified using the model order optimized over the ARX structure [43]. The whiteness of the prediction errors of HP and its mutual uncorrelation, even at zero lag, with the R series were checked in correspondence of the optimal model order [41]. 

After the identification of the model coefficients the prediction error of HP was computed as the difference between the current value of HP and its best prediction obtained by filtering the HP and R series with the estimated coefficients [43]. The variances of the prediction error were taken as the estimates of $\lambda_{ARX}^{2}$, $\lambda_{AR}^{2}$ and $\lambda_{X}^{2}$ necessary to compute $I_{HP,R}^{HP}\left(HP_{n}\right)$, $R_{HP}\left(HP_{n};HP_{n}^{-},R_{n}^{-}\right)$ and $S_{HP}\left(HP_{n};HP_{n}^{-},R_{n}^{-}\right)$. Due to normalization applied to the R and HP series $\lambda^{2}$ = 1.

### 3.6. Computation of Traditional Markers Describing Cardiorespiratory Interactions

Two traditional markers of cardiorespiratory interactions were computed, namely the respiratory sinus arrhythmia [16,17] and the strength of the relation between HP series and R at the respiratory rate [20,44]. The respiratory sinus arrhythmia was computed in the frequency domain as the power of the HP series in the high frequency (HF) band [45] and this index was labeled as HF_HP_. The HF band was defined as the range of frequencies from f_R_ − Δf_R_ to f_R_ + Δf_R_ with Δf_R_ = 0.04 Hz. Like in the case of R series power spectrum was estimated based on an AR description of the HP series [37]. The Akaike’s figure of merit was utilized to optimize the model order in the range from 8 to 14 and the Levinson-Durbin recursive method was exploited to identify the model coefficients and the variance of the white noise [37]. HF_HP_ was computed by summing the power of all spectral components whose central frequency dropped in the HF band [20]. HF_HP_ power was expressed in ms^2^. The strength of the relation between HP series and R was estimated via the squared coherence function between HP series and R [20] defined as the ratio of the square cross-spectrum modulus divided by product of the power spectra of HP and R series. The function was sampled at the maximum within the HF band and this index was termed K^2^_HP-R_(HF). By definition K^2^_HP-R_(HF) is bounded between 0 and 1 where 0 and 1 indicate minimum and maximum correlation between HP series and R in the HF band. The squared coherence function was estimated according to a bivariate AR model [41]. The model order was fixed to 10, and the coefficients of the bivariate AR model were identified via least squares approach [41].

### 3.7. Statistical Analysis

In the protocol for healthy subjects one way repeated measures analysis of variance, or Friedman repeated measures analysis of variance on ranks when appropriate, were applied to test the significance of changes of markers compared to SR. Dunnett’s test was carried out to deal with the issue of multiple comparisons. In CHF patients, paired t-test, or Wilcoxon signed rank test when appropriate, was carried out to test changes of markers compared to SR. Mann-Whitney rank sum test was used to check differences between the two groups of CHF patients in relation to age, left ventricular ejection fraction and New York Heart Association class. Fisher exact test was utilized to assess whether the two groups of CHF patients featured the same proportion of males. Linear correlation analysis of traditional markers of cardiorespiratory interactions on redundancy/synergy indexes was carried out. Pearson correlation coefficient *r* and type I error probability *p* was computed. Statistical analysis was carried out using a commercial statistical program (Sigmaplot, Systat Software, Inc, Chicago, IL, USA, version 11.0). A *p* < 0.05 was always considered statistically significant.

## 4. Results

### 4.1. Results of the Paced Breathing Protocol in Healthy Young Subjects

Table 1 reports HP mean (μ_HP_), HP variance (σ^2^_HP_), f_R_, HF_HP_ and K^2^_HP-R_(HF) as a function of the experimental condition (i.e., SR, CR10, CR15 and CR20). Paced breathing protocol decreased f_R_ compared to SR during CR10 and significantly increased f_R_ during CR20. μ_HP_ was not affected by the experimental protocol, while σ^2^_HP_ and HF_HP_ significantly increased during CR10 compared to SR. K^2^_HP-R_(HF) augmented during controlled breathing compared to SR and this result held regardless of the rate of controlled breathing. 

**Figure 3:**
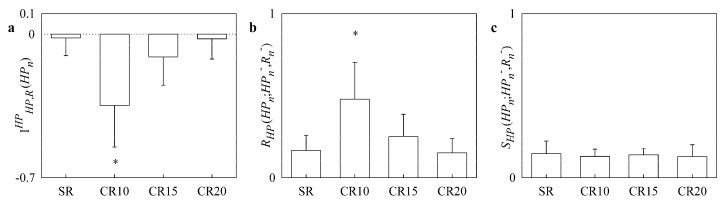
The simple bar graphs show $I_{HP,R}^{HP}\left(HP_{n}\right)$ (**a**), $R_{HP}\left(HP_{n};HP_{n}^{-},R_{n}^{-}\right)$ (**b**) and $S_{HP}\left(HP_{n};HP_{n}^{-},R_{n}^{-}\right)$ (**c**) during controlled respiration protocol in healthy young subjects as a function of the experimental condition (i.e., SR, CR10, CR15 and CR20). Values are reported as mean plus standard deviation. The symbol * indicates a significant difference versus SR with *p* < 0.05.

The simple bar graphs in Figure 3 show $I_{HP,R}^{HP}\left(HP_{n}\right)$ (Figure 3a) $R_{HP}\left(HP_{n};HP_{n}^{-},R_{n}^{-}\right)$ (Figure 3b) and $S_{HP}\left(HP_{n};HP_{n}^{-},R_{n}^{-}\right)$ (Figure 3c) as a function of the experimental condition (i.e., SR, CR10, CR15 and CR20). During CR10 $I_{HP,R}^{HP}\left(HP_{n}\right)$ significantly decreased below 0 (Figure 3a), while $R_{HP}\left(HP_{n};HP_{n}^{-},R_{n}^{-}\right)$ significantly increased (Figure 3b). Paced breathing protocol did not affect $S_{HP}\left(HP_{n};HP_{n}^{-},R_{n}^{-}\right)$ (Figure 3c).

Table 2 summarizes the results of the linear correlation analysis of traditional cardiorespiratory markers [i.e., HF_HP_ and K^2^_HP-R_(HF)] on redundancy/synergy markers [i.e., $I_{HP,R}^{HP}\left(HP_{n}\right)$, $R_{HP}\left(HP_{n};HP_{n}^{-},R_{n}^{-}\right)$ and $S_{HP}\left(HP_{n};HP_{n}^{-},R_{n}^{-}\right)$] during paced breathing protocol in healthy young subjects. Pearson correlation coefficient *r* and type I error probability *p* are reported as a function of the experimental conditions (i.e., SR, CR10, CR15 and CR20). A significant association between redundancy/synergy markers and traditional cardiorespiratory indexes was not detected systematically, thus suggesting a certain degree of independency between these two types of parameters. However, few cases of significant association, or correlation close to significance, were found in any experimental condition. When a significant association, or a correlation close to significance, was detected, *r* between $I_{HP,R}^{HP}\left(HP_{n}\right)$ and traditional cardiorespiratory markers was negative, while *r* between $R_{HP}\left(HP_{n};HP_{n}^{-},R_{n}^{-}\right)$, or $S_{HP}\left(HP_{n};HP_{n}^{-},R_{n}^{-}\right)$, and traditional cardiorespiratory parameters was positive. This finding did not depend on the selected traditional cardiorespiratory index [i.e., HF_HP_ or K^2^_HP-R_(HF)].

### 4.2. Results of the Paced and Slow Breathing Protocols in CHF Patients

The two groups of CHF patients were similar in terms of age, gender distribution and New York Heart Association class. The CHF group performing CR6 had a significantly lower left ventricular ejection fraction compared to the group undergoing CR15.

Table 3 reports the same variables as Table 1 in the group of CHF patients undergoing paced breathing. CR15 decreased significantly f_R_ and increased K^2^_HP-R_(HF). However, CR15 did not modify μ_HP_, σ^2^_HP_ and HF_HP_. Table 4 has the same structure as Table 3 but it is relevant to the group of CHF patients undergoing slow breathing. CR6 decreased significantly f_R_ but left unchanged μ_HP_, σ^2^_HP_, HF_HP_ and K^2^_HP-R_(HF).

**Figure 4:**
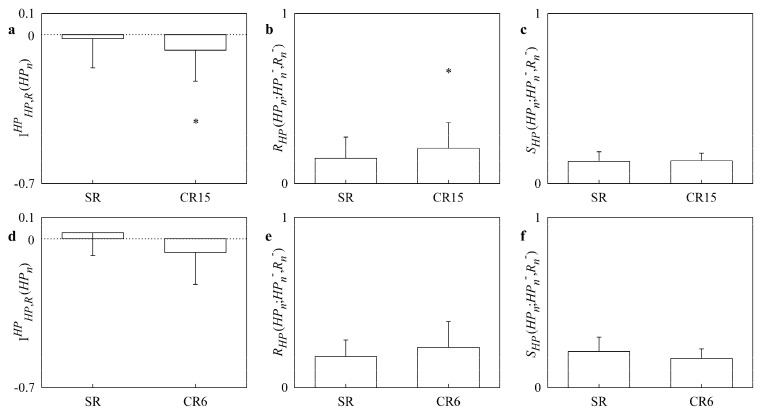
The simple bar graphs show $I_{HP,R}^{HP}\left(HP_{n}\right)$, $R_{HP}\left(HP_{n};HP_{n}^{-},R_{n}^{-}\right)$ and $S_{HP}\left(HP_{n};HP_{n}^{-},R_{n}^{-}\right)$ in CHF patients during paced breathing (a,b,c) as a function of the experimental condition (i.e., SR and CR15), and during slow breathing (d,e,f) as a function of the experimental condition (i.e., SR and CR6). Values are reported as mean plus standard deviation. The symbol * indicates a significant difference versus SR with *p* < 0.05.

Figure 4 has the same structure as Figure 3 but the upper panels (Figure 4a–c) are relevant to the CHF patients undergoing paced breathing, while the lower panels (Figure 4c–e) to CHF patients enrolled from slow breathing protocol. $I_{HP,R}^{HP}\left(HP_{n}\right)$ decreased significantly below 0 during paced breathing (Figure 4a) but was unmodified by slow breathing (Figure 4d). $R_{HP}\left(HP_{n};HP_{n}^{-},R_{n}^{-}\right)$ increased significantly during paced breathing (Figure 4b) but was unaffected by slow breathing (Figure 4e). $S_{HP}\left(HP_{n};HP_{n}^{-},R_{n}^{-}\right)$ was not affected either by paced and slow breathing protocols (Figure 4c,f).

Table 5 and Table 6 have the same structure as Table 2 but they summarize the results of the linear correlation analysis during, respectively, paced and slow breathing protocols in CHF patients. Sparse significant correlations, or correlations borderline to significance, could be detected and, when this situation was found, the sign of the correlation was the same as outlined by Table 2.

## 5. Discussion

The main findings of this study can be summarized as follows:

1. applly two approaches framed in the field of information dynamics for the assessment of redundancy/synergy in bivariate stochastic systems;
2. the two approaches were made operational in Gaussian bivariate stochastic systems describing the interactions between heart and respiration;
3. in healthy young subjects pacing respiration at a frequency slower than the spontaneous one increased redundancy of the cardiorespiratory control;
4. this effect was visible in CHF population as well even though the increase of redundancy might be limited by factors related to the efficiency of the cardiac pump;
5. redundancy/synergy parameters provided information complementary to the respiratory sinus arrhythmia and strength of the HP-R linear coupling at the respiratory rate.

### 5.1. Assessing Redundancy/Synergy in Bivariate Stochastic Systems

The methodological novelty of the study lies in stressing the possibility that redundancy/synergy can take place in bivariate dynamical systems and in highlighting the relevance of its quantification in a practical bivariate context (i.e., evaluation of cardiorespiratory control from HP series and R). In bivariate stochastic dynamical systems the redundant/synergistic term arises from the interactions of the past $X_{n}^{-}$ of the target and the past $Y_{n}^{-}$ of the driver in reducing the uncertainty associated with the present state *X_n_* of the target. Given that the necessary condition is that X exhibits internal dynamics (i.e., a dependence of *X_n_* on $X_{n}^{-}$), this redundant/synergistic term can be included into the category of interaction self-information [7,8]. Its membership to this category is supported also by the fact that this information-theoretic quantity arises from the decomposition of $S_{X}\left(X_{n}\right)$ [7]. However, Equation (13) suggests that the considered redundant/synergistic term could be included in the category of interaction cross-information as well. Two approaches for the assessment of redundancy/synergy were applied. The first approach is based on the decomposition of $P_{X}\left(X_{n}\right)$ [7,8] via Equation (16), thus calculating the net balance between synergy and redundancy, namely $I_{X,Y}^{X}\left(X_{n}\right)$, as the difference between the $P_{X}\left(X_{n}\right)$ and the sum of the information stored in *X*, namely $S_{X}\left(X_{n}\right)$, and the cross-information from *Y* to *X*, namely $C_{Y\to X}\left(X_{n}\right)$. The second approach, based on partial information decomposition framework, assesses separately redundancy and synergy as nonnegative quantities [11] with redundancy given by the minimum between $MI\left(X_{n};X_{n}^{-}\right)$ and $MI\left(X_{n};Y_{n}^{-}\right)$ [9], and synergy given by Equation (18). Indexes of the net balance between synergy and redundancy can be interpreted in terms of the ability of the source Y to enhance (positive balance meaning prevalent synergy) or inhibit (negative balance meaning prevalent redundancy) the information storage in X as suggested by Equation (10). Equivalently, the net balance between synergy and redundancy can be interpreted in terms of the ability of the past of *X* to enhance (positive balance meaning prevalent synergy) or inhibit (negative balance meaning prevalent redundancy) the cross-information from *Y* to *X* as suggested by Equation (13). Alternatively, we can say that a prevalent synergy occurs when the information about *X_n_* jointly carried by $X_{n}^{-}$ and $Y_{n}^{-}$ [i.e., the $P_{X}\left(X_{n}\right)$] is larger than the separate contributions of $X_{n}^{-}$ and $Y_{n}^{-}$, quantified by $S_{X}\left(X_{n}\right)$ and $C_{Y\to X}\left(X_{n}\right)$ respectively. The opposite occurs in presence of prevalent redundancy. The approach assessing redundancy and synergy as nonnegative quantities preserves the interpretation linked to the balance between synergy and redundancy but it allows one to discover whether situations characterized by the contemporaneous rise or decrease of both quantities might happen.

### 5.2. On the Relevance of Assessing Redundancy/Synergy of the Cardiorespiratory Interactions

R has a profound impact on HP dynamics and the most evident effect is the respiratory sinus arrhythmia (i.e., heart rate increases during inspiration and decreases during expiration). A series of physiological mechanisms are responsible for the HP-R relation and all are subsumed with the term *cardiorespiratory coupling* [46]. During inspiration venous return to the right atrium increases as a result of the decreased intrathoracic pressure, while the blood pools in the pulmonary circulation due to the augmented pulmonary resistance, thus reducing left ventricular filling and consequently, stroke volume [47]. The reduction of the stroke volume leads to a modification of arterial pressure that might be sensed by baroreceptors and, via the fast vagal arm of the cardiac baroreflex, induces, in turn, HP changes paralleling those of arterial pressure [48] and this response might occur even within the same HP where the arterial pressure drop is observed [38,39]. The opposite phenomena are observed during expiratory phase inducing rhythmical HP fluctuations at the respiratory rate known as respiratory sinus arrhythmia [17]. However, the HP-R link cannot be exclusively explained by the fast baroreflex-mediated response to modifications of arterial pressure driven by changes of the venous return [49]. Indeed, if this was the case, HP variations would always lag behind arterial pressure changes at the respiratory rate, while, conversely, at supine rest it was observed that HP might lead arterial pressure variations [50] with a directionality of the interactions from HP to SAP [51]. Phase advancements of HP with respect to arterial pressure at the respiratory rate are compatible with the presence of a direct influence of R on HP variability mediated by the action of respiratory centers [52] modulating activity and responsiveness of vagal motoneurons [16] and, thus, HP. Also, the Bainbridge reflex accounting for the bradycardic response to the solicitation of atrial stretch receptors owing to the increased venous return during inspiration [53] and Hering-Breuer reflex accounting for the response of pulmonary stretch receptors activated during lung inflation and acting on respiratory centers and autonomic outflows via afferent neural pathways [54] contribute to shape the cardiorespiratory coupling. The HP-R coupling is not exclusively mediated by changes of the vagal outflow at the respiratory rate but also by modifications of the sympathetic drive resulting from the inhibition of sympathetic burst in the late half of inspiration and initial half of expiration [55] and by the activation of the sympathetic arm of the baroreflex facilitating sympathetic bursts when the within-breath fluctuations of arterial pressure reach the nadir [56,57].

All the mechanisms responsible for setting the HP-R link are empowered and made more effective by paced respiration, especially at slow breathing rate. Indeed, while decreasing the breathing rate, baroreflex sensitivity increases [58,59], atrial and pulmonary stretch receptors are more solicited, thus gating more efficiently respiratory centers and autonomic activity [55], and the sinus node transfer function linking vagal activity to HP variability exhibits larger gains [20,60]. The final result is an increased respiratory sinus arrhythmia, a more powerful coupling between HP and arterial pressure at the respiratory rate, and tighter HP-R coordination while decreasing the breathing rate [59,60,61,62]. The course of traditional cardiorespiratory markers computed in this study confirmed these observations.

The common feature of the studies mentioned above is the attempt to disentangle the genuine contribution of one mechanism in relation to the others, while disregarding effects that are mostly related to their common actions. As a consequence, they were very powerful in describing the effect of controlled breathing maneuver over a peculiar mechanism (e.g., the augmented cardiac baroreflex sensitivity while decreasing the breathing rate), but they were useless in describing redundant/synergistic influences resulting from their shared actions. Conversely, the present study does not focus on a specific mechanism but on the redundant/synergistic aspects of the cardiorespiratory interactions. The approach was made operational under the hypothesis of Gaussianity thanks to the exploitation of model-based information-theoretic frameworks. However, the same quantities can be computed more generally according to a model-free approach [44] by following the definition given in Section 2.2. The remarkable feature of this approach is the possibility to assess cardiorespiratory redundancy/synergy from a bivariate set of data exclusively composed by HP variability and R signal. The sole exploitation of the HP variability and R signal enlarges the possibility to assess markers of cardiorespiratory redundancy/synergy because the HP series can be easily derived from the ECG [45] and R can be easily monitored, for instance, from movements of the thorax recorded via a respiratory belt or even extracted from amplitude modulations of the electrocardiogram reflecting respiratory-related cardiac axis movements [63].

### 5.3. Paced Breathing Increases Redundancy of Cardiorespiratory Control in Healthy Young Subjects and CHF Patients

The original result of this study is that breathing at a rate slower than the spontaneous frequency increases cardiorespiratory redundancy in healthy young subjects. This means that cardiac and respiratory controls, when jointly observed, contribute to the information carried by HP with less information than the sum of information due to their separate actions. Remarkably, this conclusion held in chronic pathological individuals, such as CHF patients, and did not depend on the framework utilized for the analysis (i.e., predictive or partial information decomposition approaches). This effect is likely to be related to the strengthening of all the mechanisms recalled in Section 5.2. Indeed, all these mechanisms are still present under spontaneous breathing but their influence is limited because their action is dispersed within the myriad of different control reflexes and feedforward pathways governing HP dynamics [64]. Conversely, the solicitation imposed by paced breathing with a frequency significantly slower than the spontaneous one leads to the intensification of the action of these mechanisms and, given that some redundancy is present during SR, their redundant action in targeting HP is empowered. Redundancy returns to be limited and comparable to SR if the frequency of the controlled respiration is comparable or higher than that of spontaneous respiration, likely because the entity of the solicitation is inadequate or inferior to that observed during natural breathing rate. In CHF patients the increase of redundancy is more evident in the group undergoing paced than slow breathing. Since CHF patients undergoing slow breathing protocol had left ventricular ejection fraction significantly lower than those enrolled for paced breathing protocol at 15 breaths/min, we suggest that the increase of redundancy associated with paced breathing becomes significant only whether the cardiac pump is sufficiently efficient. If, conversely, the left ventricular ejection fraction is too limited, solicitations coming from an empowered respiratory drive might become less effective (e.g., an impaired cardiac pump limits the changes of stroke volume at the respiratory rate, thus reducing the relevance of the cardiac baroreflex solicitations). An alternative interpretation of the absence of a significant increase of redundancy during slow breathing in CHF patients is that this protocol, by imposing a paced respiration at the resonance frequency of the cardiac baroreflex control (i.e., about 0.1 Hz) [65], leads to an entrainment between baroreflex mechanisms and respiration that could limit the ability of the method to provide a reliable description of the cardiorespiratory interactions due to the reduction of dimensionality of the bivariate system (i.e., X and Y systems can be considered no longer as separate entities).

### 5.4. Correlation of Redundancy/Synergy Markers with Traditional Cardiorespiratory Indexes

The correlation analysis detected few cases of significant association between redundancy/synergy markers and traditional indexes of cardiorespiratory interactions, such as the respiratory sinus arrhythmia and the peak squared coherence between HP series and R in HF band. When association was significant, or borderline to significance, the sign of the correlation of traditional cardiorespiratory markers on indexes of synergy and redundancy computed separately as nonnegative quantities was positive. This result suggests that an increased vagal modulation, producing a rise of respiratory sinus arrhythmia and a stronger linear relation between HP series and R, might lead to the contemporaneous and separate increase of both redundancy and synergy. Given that few cases of significant association between the net synergy/redundancy balance and traditional markers of cardiorespiratory interactions were detected as well but the sign of the correlation was negative, we suggest that situations characterized by an increase of vagal modulation led to a greater augmentation of redundancy than synergy. However, the sparse nature of the correlations (i.e., a significant correlation between redundancy/synergy parameters and traditional cardiorespiratory markers was not detected systematically in any protocol and any experimental condition) indicates that indexes assessing redundancy/synergy carry complementary information to classical markers and cannot be simply considered proxies of them. 

## 6. Conclusions

We applied a bivariate approach framed in the field of information dynamics to assess redundancy/synergy between heart and respiration. The approach reveals that a breathing protocol at a frequency slower than the spontaneous one increased redundancy of cardiorespiratory control and this effect was observed in healthy subjects as well as in chronic pathological individuals such as CHF patients, thus quantifying a peculiar aspect of the cardiorespiratory coordination that cannot be addressed by indexes traditionally utilized in the evaluation of cardiorespiratory coupling. Given the minimal number of involved signals the technique allows the computation of the redundancy of the cardiorespiratory control in practical contexts where HP variability and R can be recorded directly or derived from other signals. Moreover, given that redundancy/synergy markers are mostly uncorrelated with more traditional indexes of cardiorespiratory interactions, such as the respiratory sinus arrhythmia and the degree of cardiorespiratory linear coupling, they deserve to be computed in any practical applications devoted to the monitoring of cardiorespiratory control both in healthy subjects and pathological patients. Future studies should be focused on the clarification of the clinical impact of the quantification of synergy/redundancy in cardiorespiratory control by linking the proposed parameters to variables directly associated with clinical outcome and on the elucidation of neural integration mechanisms underlying cardiorespiratory redundancy/synergy by assessing the proposed indexes in experimental models in which cardiorespiratory control is modified in a well-controlled manner via e.g., pharmacological challenges or graded stimuli.

## Figures and Tables

**Figure 1:**
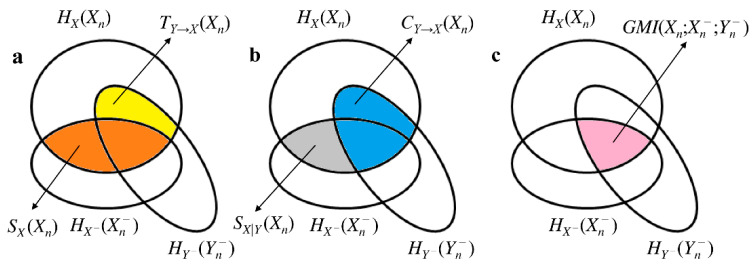
Mnemonic Venn diagram of the information-theoretic quantities listed in (Equations (1)–(7)). The three intercepting ellipses represent $H_{X}\left(X_{n}\right)$, $H_{X^{-}}\left(X_{n}^{-}\right)$ and $H_{Y^{-}}\left(Y_{n}^{-}\right)$. The quantities $S_{X}\left(X_{n}\right)$ (orange area), $T_{Y\to X}\left(X_{n}\right)$ (yellow area), $S_{X|Y}\left(X_{n}\right)$ (grey area) and $C_{Y\to X}\left(X_{n}\right)$ (blue area) are shown in (**a**) and (**b**). $S_{X}\left(X_{n}\right)$, $T_{Y\to X}\left(X_{n}\right)$, $S_{X|Y}\left(X_{n}\right)$ and $C_{Y\to X}\left(X_{n}\right)$ contribute to the definition of $GMI\left(X_{n};X_{n}^{-};Y_{n}^{-}\right)$ (**c**, pink area). The sum of the orange and yellow areas is $P_{X}\left(X_{n}\right)$ as well as the sum of the grey and blue areas.

**Table 1:** Traditional markers derived from HP and R series during paced breathing in healthy young subjects.

	SR	CR10	CR15	CR20
µ_HP_ [ms]	1009.86 ± 167.92	989.33 ± 156.82	1022.53 ± 161.94	1027.95 ± 162.37
σ^2^_HP_ [ms^2^]	3368.30 ± 2622.13	4784.17 ± 3355.86 *	3704.77 ± 3090.59	2812.66 ± 2158.02
f_R_ [Hz]	0.260 ± 0.031	0.210 ± 0.049 *	0.250 ± 0.026	0.310 ± 0.024 *
HF_HP_ [ms^2^]	1405.41 ± 1555.21	2856.10 ± 2969.00 *	1891.23 ± 1906.98	1140.68 ± 1205.75
K^2^_HP-R_(HF)	0.85 ± 0.11	0.93 ± 0.08 *	0.96 ± 0.04 *	0.92 ± 0.05 *

HP = heart period; R = respiration; μ_HP_ = HP mean; σ^2^_HP_ = HP variance; f_R_ = R frequency; HF = high frequency; HF_HP_ = HF power of the HP series expressed in absolute units; K^2^_HP-R_(HF) = peak value of squared coherence between HP and R series in the HF band; SR = at rest in supine position during spontaneous respiration; CR10 = at rest in supine position during controlled respiration at 10 breaths/minute; CR15 = at rest in supine position during controlled respiration at 15 breaths/minute; CR20 = at rest in supine position during controlled respiration at 20 breaths/minute. Values are reported as mean ± standard deviation. The symbol * indicates a significant difference versus SR with *p* < 0.05.

**Table 2:** Results of the correlation analysis between redundancy/synergy parameters and traditional markers of cardiorespiratory interactions during paced breathing in healthy young subjects.

	Marker	HF_HP_	K^2^_HP-R_(HF)
	$I_{HP,R}^{HP}\left(HP_{n}\right)$	−0.438; 6.04 × 10^−2^ #	−0.142; 5.61 × 10^−1^
SR	$R_{HP}\left(HP_{n};HP_{n}^{-},R_{n}^{-}\right)$	0.556; 1.34 × 10^−2^ *	0.450; 5.32 × 10^−2^ #
	$S_{HP}\left(HP_{n};HP_{n}^{-},R_{n}^{-}\right)$	0.191; 4.34 × 10^−1^	0.395; 9.41 × 10^−2^
	$I_{HP,R}^{HP}\left(HP_{n}\right)$	−0.099; 6.86 × 10^−1^	−0.415; 7.72 × 10^−2^
CR10	$R_{HP}\left(HP_{n};HP_{n}^{-},R_{n}^{-}\right)$	0.041; 8.68 × 10^−1^	0.453; 5.15 × 10^−2^ #
	$S_{HP}\left(HP_{n};HP_{n}^{-},R_{n}^{-}\right)$	−0.243; 3.16 × 10^−1^	0.398; 9.17 × 10^−2^
	$I_{HP,R}^{HP}\left(HP_{n}\right)$	−0.556; 1.35 × 10^−2^ *	−0.431; 6.52 × 10^−2^ #
CR15	$R_{HP}\left(HP_{n};HP_{n}^{-},R_{n}^{-}\right)$	0.452; 5.20 × 10^−2^ #	0.584; 8.69 × 10^−3^ *
	$S_{HP}\left(HP_{n};HP_{n}^{-},R_{n}^{-}\right)$	−0.339; 9.02 × 10^−2^	0.497; 3.04 × 10^−2^ *
	$I_{HP,R}^{HP}\left(HP_{n}\right)$	−0.521; 2.23 × 10^−2^ *	0.213; 3.81 × 10^−1^
CR20	$R_{HP}\left(HP_{n};HP_{n}^{-},R_{n}^{-}\right)$	0.328; 1.70 × 10^−1^	0.064; 7.93 × 10^−1^
	$S_{HP}\left(HP_{n};HP_{n}^{-},R_{n}^{-}\right)$	−0.315; 1.89 × 10^−1^	0.365; 1.25 × 10^−1^

HP = heart period; R = respiration; HF = high frequency; HF_HP_ = HF power of the HP series expressed in absolute units; K^2^_HP-R_(HF) = peak value of squared coherence between HP and R series in the HF band; $I_{HP,R}^{HP}\left(HP_{n}\right)$, $R_{HP}\left(HP_{n};HP_{n}^{-},R_{n}^{-}\right)$ and $S_{HP}\left(HP_{n};HP_{n}^{-},R_{n}^{-}\right)$ = markers of redundancy/synergy; SR = at rest in supine position during spontaneous respiration; CR10 = at rest in supine position during controlled respiration at 10 breaths/minute; CR15 = at rest in supine position during controlled respiration at 15 breaths/minute; CR20 = at rest in supine position during controlled respiration at 20 breaths/minute. Pearson correlation coefficient and type I error probability were separated by semicolon. The symbol * indicates *p* < 0.05. The symbol # indicates a borderline significance (i.e., *p* close to significance).

**Table 3:** Traditional markers derived from HP and R series during paced breathing in CHF patients.

	SR	CR15
µ_HP_ [ms]	934.73 ± 121.12	931.85 ± 125.90
σ^2^_HP_ [ms^2^]	710.46 ± 762.30	626.69 ± 538.63
f_R_ [Hz]	0.289 ± 0.053	0.239 ± 0.024 *
HF_HP_ [ms^2^]	162.74 ± 204.04	203.38 ± 162.52
K^2^_HP-R_ (HF)	0.89 ± 0.09	0.97 ± 0.03 *

HP = heart period; R = respiration; μ_HP_ = HP mean; σ^2^_HP_ = HP variance; f_R_ = R frequency; HF = high frequency; HF_HP_ = HF power of the HP series expressed in absolute units; K^2^_HP-R_(HF) = peak value of squared coherence between HP and R series in the HF band; SR = at rest in supine position during spontaneous respiration; CR15 = at rest in supine position during controlled respiration at 15 breaths/minute. Values are reported as mean ± standard deviation. The symbol * indicates a significant difference versus SR with *p* < 0.05.

**Table 4:** Traditional markers derived from HP and R series during slow breathing in CHF patients.

	SR	CR6
µ_HP_ [ms]	816.36 ± 169.53	818.83 ± 155.94
σ^2^_HP_ [ms^2^]	184.44 ± 179.38	339.90 ± 415.33
f_R_ [Hz]	0.292 ± 0.065	0.123 ± 0.018 *
HF_HP_ [ms^2^]	24.89 ± 19.59	105.71 ± 168.31
K^2^_HP-R_ (HF)	0.88 ± 0.14	0.94 ± 0.07

HP = heart period; R = respiration; μ_HP_ = HP mean; σ^2^_HP_ = HP variance; f_R_ = R frequency; HF = high frequency; HF_HP_ = HF power of the HP series expressed in absolute units; K^2^_HP-R_(HF) = peak value of squared coherence between HP and R series in the HF band; SR = at rest in supine position during spontaneous respiration; CR6 = at rest in supine position during controlled respiration at 6 breaths/minute. Values are reported as mean ± standard deviation. The symbol * indicates a significant difference versus SR with *p* < 0.05.

**Table 5:** Results of the correlation analysis between redundancy/synergy parameters and traditional markers of cardiorespiratory interactions during paced breathing in CHF patients.

	Marker	HF_HP_	K^2^_HP-R_(HF)
	$I_{HP,R}^{HP}\left(HP_{n}\right)$	−0.454; 4.42 × 10^−2^ *	−0.012; 9.58 × 10^−1^
SR	$R_{HP}\left(HP_{n};HP_{n}^{-},R_{n}^{-}\right)$	0.484; 3.06 × 10^−2^ *	0.245; 2.97 × 10^−1^
	$S_{HP}\left(HP_{n};HP_{n}^{-},R_{n}^{-}\right)$	−0.047; 8.45 × 10^−1^	0.516; 1.98 × 10^−2^ *
	$I_{HP,R}^{HP}\left(HP_{n}\right)$	−0.345; 1.37 × 10^−1^	−0.401; 7.95 × 10^−2^
CR15	$R_{HP}\left(HP_{n};HP_{n}^{-},R_{n}^{-}\right)$	0.348; 1.33 × 10^−1^	0.518; 1.93 × 10^−2^ *
	$S_{HP}\left(HP_{n};HP_{n}^{-},R_{n}^{-}\right)$	0.051; 8.32 × 10^−1^	0.441; 5.16 × 10^−2^ #

HP = heart period; R = respiration; HF = high frequency; HF_HP_ = HF power of the HP series expressed in absolute units; K^2^_HP-R_(HF) = peak value of squared coherence between HP and R series in the HF band; $I_{HP,R}^{HP}\left(HP_{n}\right)$, $R_{HP}\left(HP_{n};HP_{n}^{-},R_{n}^{-}\right)$ and $S_{HP}\left(HP_{n};HP_{n}^{-},R_{n}^{-}\right)$ = markers of redundancy/synergy; SR = at rest in supine position during spontaneous respiration; CR15 = at rest in supine position during paced breathing at 15 breaths/minute. Pearson correlation coefficient and type I error probability were separated by semicolon. The symbol * indicates *p* < 0.05. The symbol # indicates a borderline significance (i.e., *p* close to significance).

**Table 6:** Results of the correlation analysis between redundancy/synergy parameters and traditional markers of cardiorespiratory interactions during slow breathing in CHF patients.

	Marker	HF_HP_	K^2^_HP-R_(HF)
	$I_{HP,R}^{HP}\left(HP_{n}\right)$	−0.212; 5.83 × 10^−1^	0.223; 5.64 × 10^−1^
SR	$R_{HP}\left(HP_{n};HP_{n}^{-},R_{n}^{-}\right)$	0.306; 4.23 × 10^−1^	0.339; 2.97 × 10^−1^
	$S_{HP}\left(HP_{n};HP_{n}^{-},R_{n}^{-}\right)$	0.074; 8.49 × 10^−1^	0.740; 2.271 × 10^−2^ *
	$I_{HP,R}^{HP}\left(HP_{n}\right)$	−0.258; 5.03 × 10^−1^	−0.275; 4.74 × 10^−1^
CR6	$R_{HP}\left(HP_{n};HP_{n}^{-},R_{n}^{-}\right)$	0.303; 4.29 × 10^−1^	0.458; 2.15 × 10^−1^
	$S_{HP}\left(HP_{n};HP_{n}^{-},R_{n}^{-}\right)$	0.130; 7.38 × 10^−1^	0.504; 1.66 × 10^−1^

HP = heart period; R = respiration; HF = high frequency; HF_HP_ = HF power of the HP series expressed in absolute units; K^2^_HP-R_(HF) = peak value of squared coherence between HP and R series in the HF band; $I_{HP,R}^{HP}\left(HP_{n}\right)$, $R_{HP}\left(HP_{n};HP_{n}^{-},R_{n}^{-}\right)$ and $S_{HP}\left(HP_{n};HP_{n}^{-},R_{n}^{-}\right)$ = markers of redundancy/synergy; SR = at rest in supine position during spontaneous respiration; CR6 = at rest in supine position during slow breathing at 6 breaths/minute. Pearson correlation coefficient and type I error probability were separated by semicolon. The symbol * indicates *p* < 0.05.
